# Supplementary material for: Can Glycosylation Mask the Detection of MHC Expressing p53 Peptides by T Cell Receptors?
Source: Biomolecules. 2021 Jul 19;11(7):1056. doi: 10.3390/biom11071056 (PMC8301869; doi:10.3390/biom11071056)
Supplement: Supplementary file 1 [file biomolecules-11-01056-s001.zip › biomolecules-1245424-supplementary.pdf]

Article

Can Glycosylation Mask the Detection of MHC Expressing p53 Peptides by T Cell Receptors?

Thanh Binh Nguyen [1], David P. Lane [2] and Chandra S. Verma [1,3,4,*]

[1] Division of Biomolecular Structure to Mechanism, Bioinformatics Institute, Agency for Science, Technology and Research (A*STAR), Singapore 138671, Singapore; nguyenbinhchem@gmail.com
[2] p53 Laboratory, Agency for Science, Technology and Research (A*STAR), Singapore 138648, Singapore; dplane@imcb.a-star.edu.sg
[3] School of Biological Sciences, College of Science, Nanyang Technological University, Singapore 637551, Singapore
[4] Department of Biological Sciences, Faculty of Science, National University of Singapore, Singapore 117543, Singapore
* Correspondence: chandra@bii.a-star.edu.sg; Tel.: +65-6478-8273; Fax: +65-6478-9048

Table S1. The lifetime of hbond interactions between A24 and TCR in the A24-peptide-TCR where the peptides are p161, Galp161, and Glcp161.

**A24-p161-TCR**						
**A24**			**TCR**			**Lifetime (%)**
**Residue Name**	**Residue Number**	**Atom Number**	**Residue Name**	**Residue Number**	**Atom Number**	
GLU	62	OE1	GLN	a-94	NE2	18
LYS	66	NZ	GLN	a-94	OE1	28
GLN	72	O	ASN	b-51	ND2	32
GLN	72	NE2	ASN	b-51	OD1	12
GLN	72	NE2	VAL	b-52	O	46
GLN	72	NE2	ASP	b-56	OD1	14
THR	73	OG1	ASN	b-51	ND2	56
GLN	155	NE2	TYR	a-51	OH	31
GLN	155	NE2	SER	b-97	OG	29
GLN	155	OE1	HID	b-98	N	51
GLU	62	OE1	LYS	b-57	NZ	28
LYS	66	NZ	ASP	b-56	OD1	19
LYS	68	NZ	ASP	b-56	OD2	25
GLU	161	OE2	ARG	a-28	NH2	23
ASP	166	OD2	ARG	a-28	NH2	48
**A24-Galp161-TCR**						
GLU	62	OE1	GLN	a-94	NE2	21
GLY	65	O	ASN	b-51	ND2	13
LYS	66	NZ	GLN	a-94	O	26
HIE	70	NE2	GLY	a-95	O	31
GLN	72	NE2	VAL	b-52	O	20
GLU	154	OE1	SER	a-32	N	21
GLU	154	OE1	SER	a-52	OG	23
GLN	155	OE1	SER	a-32	OG	36
GLN	155	NE2	SER	a-32	OG	20
GLN	155	NE2	HID	b-98	ND1	13
ARG	157	NH1	ARG	a-28	O	38
THR	163	OG1	GLN	a-31	NE2	24
THR	163	OG1	GLN	a-94	OE1	18
GLU	58	OE1	LYS	b-57	NZ	13
GLU	62	OE1	LYS	b-57	NZ	33
GLU	154	OE1	LYS	a-67	NZ	17

GLU	161	OE1	LYS	a-67	NZ	62
ASP	166	OD1	ARG	a-28	NH2	100
GLU	173	OE1	ARG	a-28	NH1	34
**A24-Glcp161-TCR**						
GLN	72	O	ASN	b-51	ND2	33
GLU	76	OE2	ASN	b-51	ND2	33
GLU	154	OE1	SER	a-52	OG	34
GLN	155	OE1	GLN	a-31	NE2	11
GLN	156	NE2	SER	a-32	OG	34
GLN	72	NE2	GLU	b-29	OE2	10
GLN	72	NE2	ASP	b-55	OD1	27
GLN	156	NE2	SER	b-97	OG	12
GLN	156	NE2	HID	b-98	NE2	22
GLU	154	OE1	LYS	a-67	NZ	16
ASP	166	OD1	ARG	a-28	NH2	33
LYS	68	NZ	ASP	b-56	OD2	27

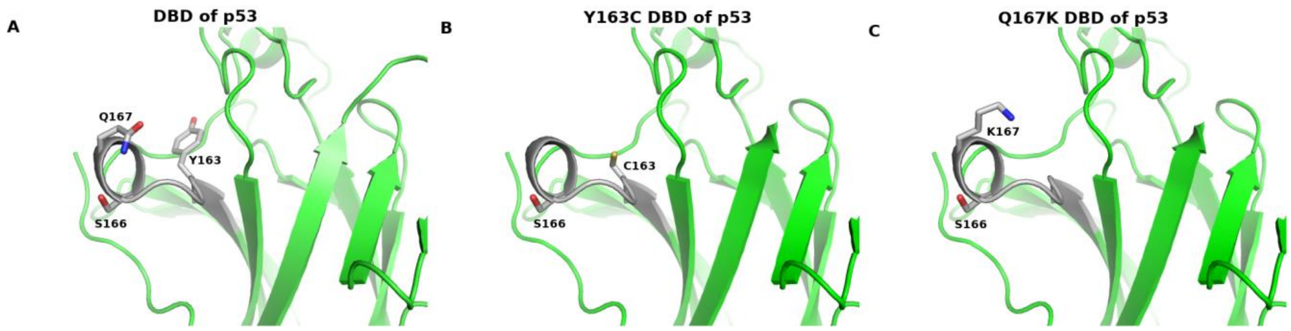

Figure S1. The DBD of p53 (green cartoon) taken from a monomer of its dimer of dimers complex with two fragments of decameric DNA (PDB ID: 2AHI, resolution 1.8 Å). The wild type protein is shown in (**A**) with the two sites of mutations (Y163 and Q167), the region 161–169 corresponding to p161 is shown in grey ribbon and the site of glycosylation S166 is shown in sticks. (**B**) the mutation Y163C is shown in sticks and it is clear that it does not occlude S166; (**C**) the mutation Q167K is shown in sticks and it is clear that it does not occlude S166; p161 is highlighted in grey; C atoms are shown in grey, Oxygen atoms are shown in red and Nitrogen atoms are shown in blue.

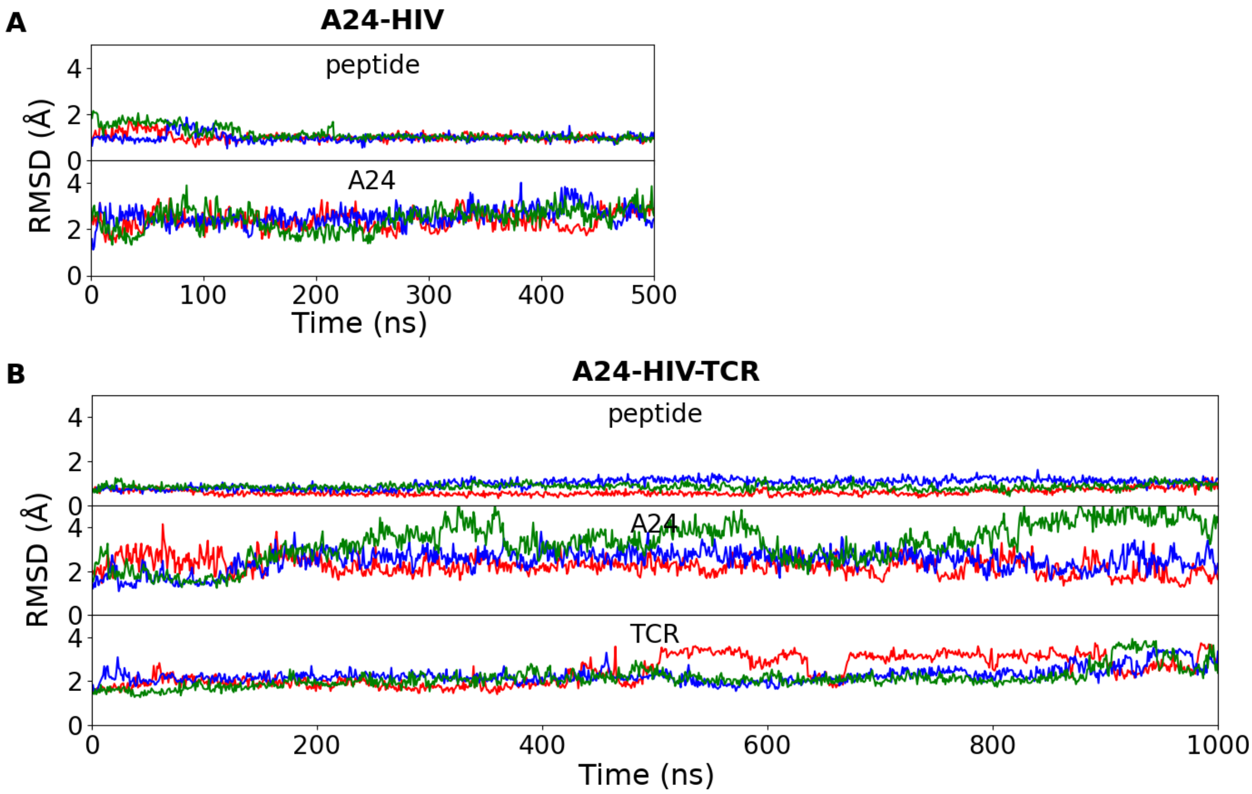

Figure S2. C$^{\alpha}$ RMSD values of the HIV peptide in complex with A24 in the (**A**) absence and (**B**) presence of TCR over 500 ns and 1000 ns of MD simulations, respectively. The RMSD values shown in Å correspond to different parts of the complexes; peptide (first row), A24 (second row) and the α and β chains TCR (third row); red, blue, and green colors represent the different replicas.

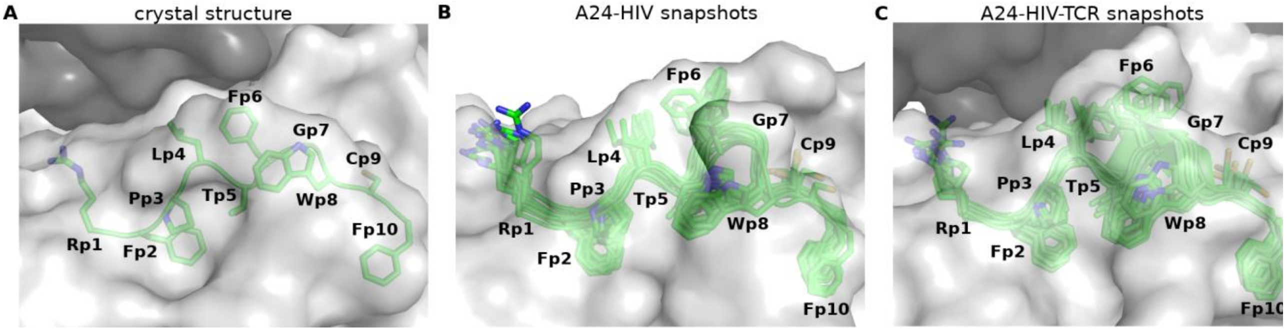

Figure S3. (**A**) Location of the HIV peptide in the crystal structure of the A24-HIV-TCR complex (PDB ID: 3VXU, resolution 2.7 Å); (**B**) Nine snapshots from the MD simulations of the binary complex of A24 with the HIV peptide (snapshots taken at 300, 400, and 500 ns of each MD simulation from the triplicate); and (**C**) Nine snapshots from the MD simulations of the ternary complex of A24 with the HIV peptide and TCR (snapshots taken at 800, 900, and 1000 ns of each MD simulation from the triplicate); A24 is in grey surface, TCR is in dark grey surface and HIV peptide is in green sticks, with Oxygen atoms in red and Nitrogen atoms in blue.

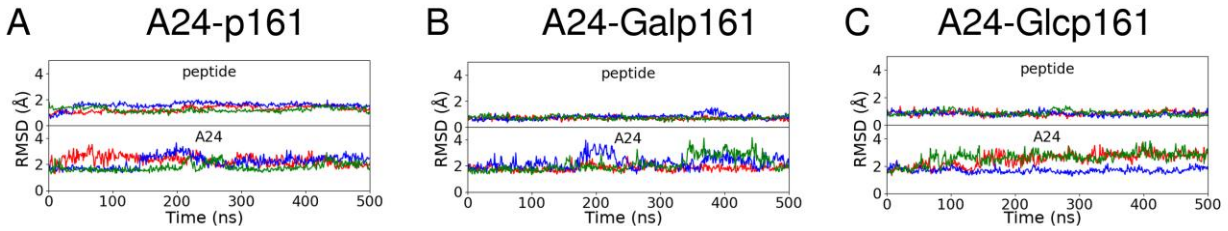

Figure S4. C^{α} RMSD values of the A24-peptide complexes where the peptides are (**A**) p161, (**B**) Galp161, and (**C**) Glcp161 during 500 ns triplicate MD simulations. The RMSD values correspond to different parts of the A24-peptide complexes, namely, all residues of the peptide (first row), and all residues of A24 (second row); red, blue, and green colors represent the different replicas.

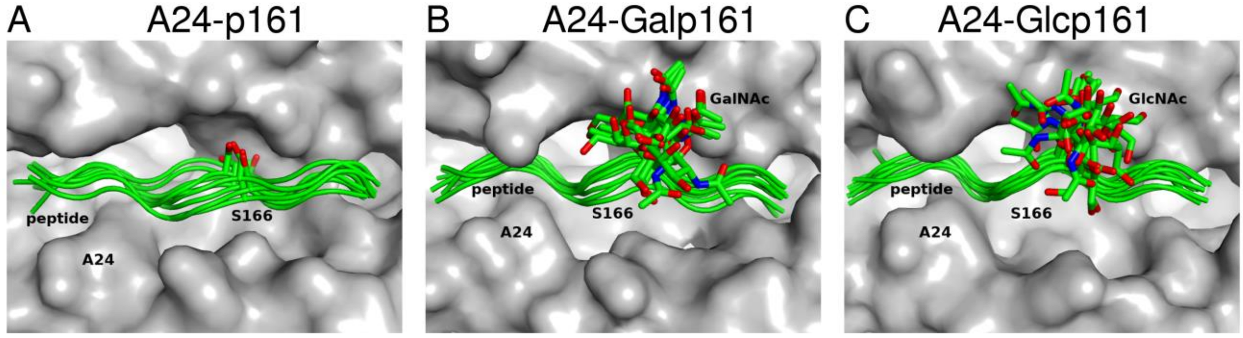

Figure S5. Conformations sampled by the p53 peptides complexed to A24 during the MD simulations of the binary A24-peptide complexes: (**A**) p161, (**B**) Galp161, and (**C**) Glcp161; Nine snapshots of the peptide are shown in each panel, with the conformations extracted at 300, 400, and 500 ns of each of the triplicate MD simulations. A24 is in grey surface, p53 peptide is in green ribbon, residue S166 and the sugars are shown in sticks with Oxygen atoms in red and Nitrogen atoms in blue.

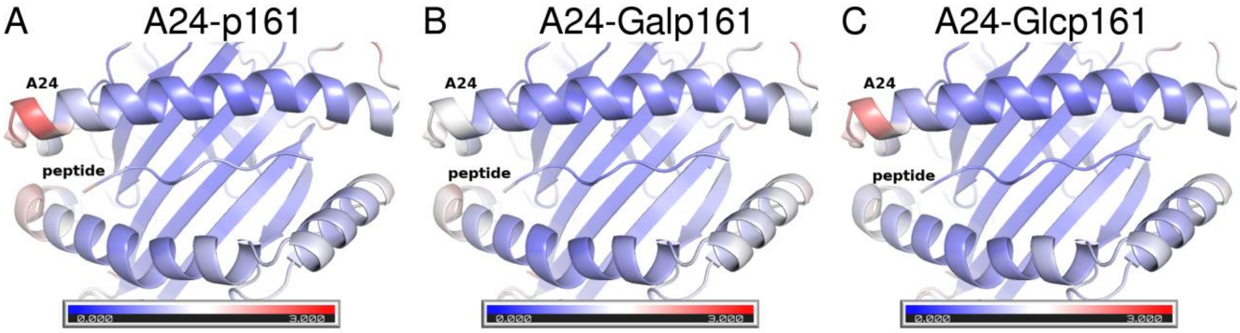

Figure S6. C^{α} RMSF of the binary complexes of A24 and the p161 peptides: peptides are (**A**) p161, (**B**) Galp161, and (**C**) Glcp161 during the last 200 ns of the MD simulations. RMSF values are coloured from very low (blue) to 3 Å (red).

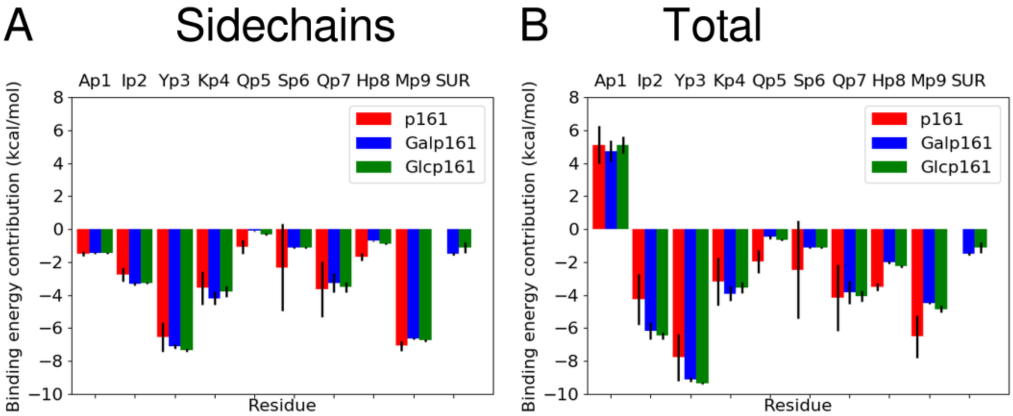

Figure S7. The contributions of (**A**) sidechains and (**B**) both sidechains and backbone atoms of the peptide residues to the total binding energies with A24 during the last 200 ns of the 500 ns triplicate MD simulations. Red, blue and green colors represent p161, Galp161, and Glcp161 respectively. Standard deviations are shown in error bars.

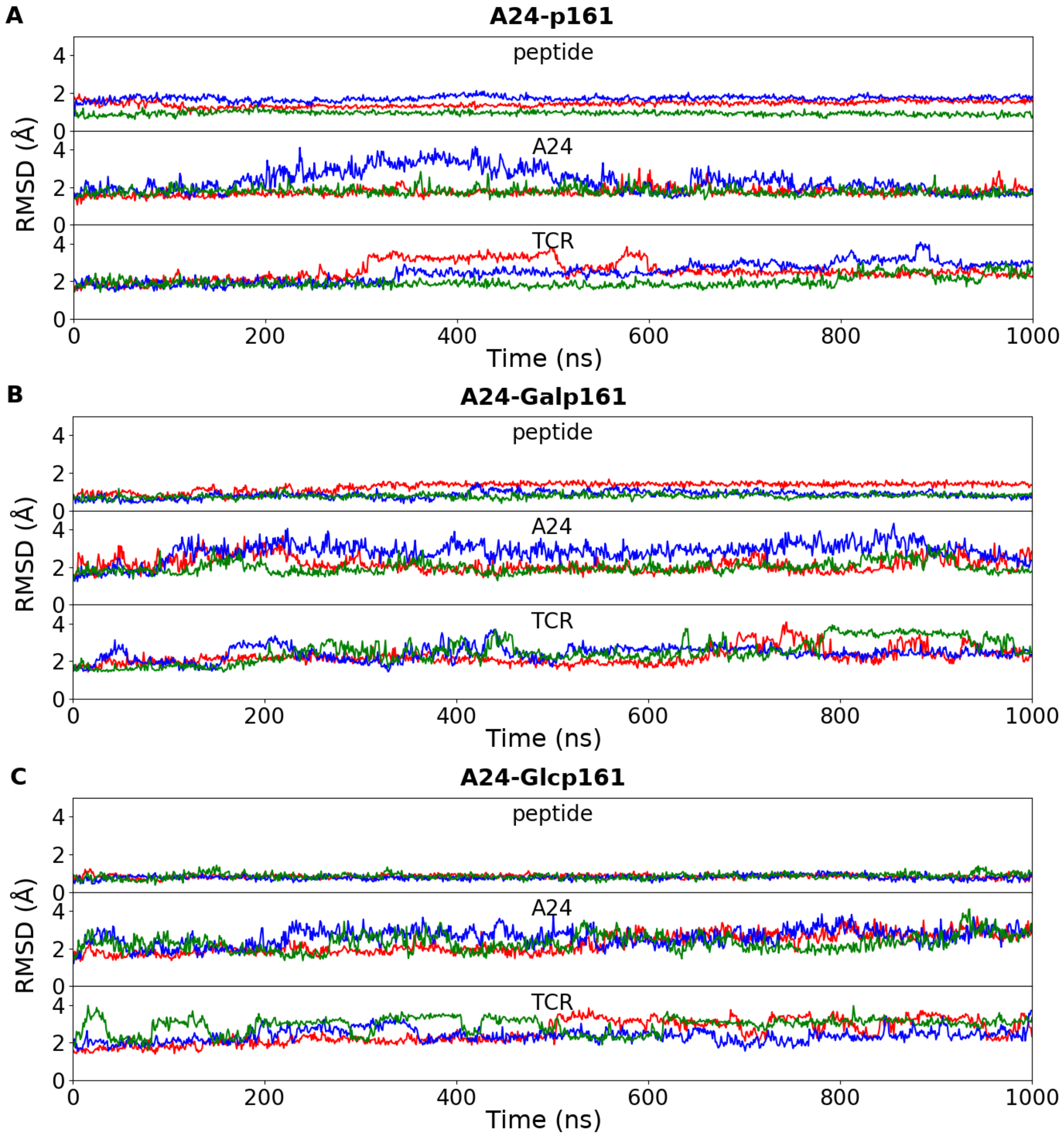

Figure S8. Cα RMSD values of the ternary A24-peptide-TCR complexes during the triplicate 1000 ns MD simulations for: (**A**) A24-p161-TCR, (**B**) A24-Galp161-TCR, and (**C**) A24-Glcp161-TCR. In each of the 3 panels, the RMSD trace corresponds to the peptide (first row), A24 (second row), and TCR (third row). Red, blue, and green colors represent different replicas.

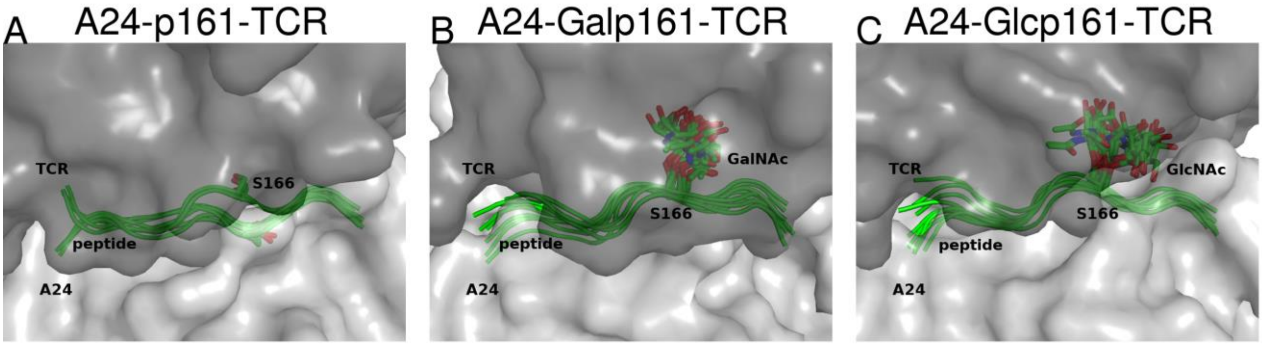

Figure S9. Conformations of the p53 peptides sampled during the MD simulations of the ternary A24-peptide-TCR complexes: (**A**) A24-p161-TCR, (**B**) A24-Galp161-TCR, and (**C**) A24-Glcp161-TCR. Each panel shows nine structural snapshots of the peptides extracted at 800, 900, and 1000 ns of each of the triplicate MD simulations. A24 is in grey surface, TCR is in dark grey surface and p53 peptide is in green ribbons. Residue S166 and its glycosylations are shown in sticks with Oxygen atoms in red and Nitrogen atoms in blue.

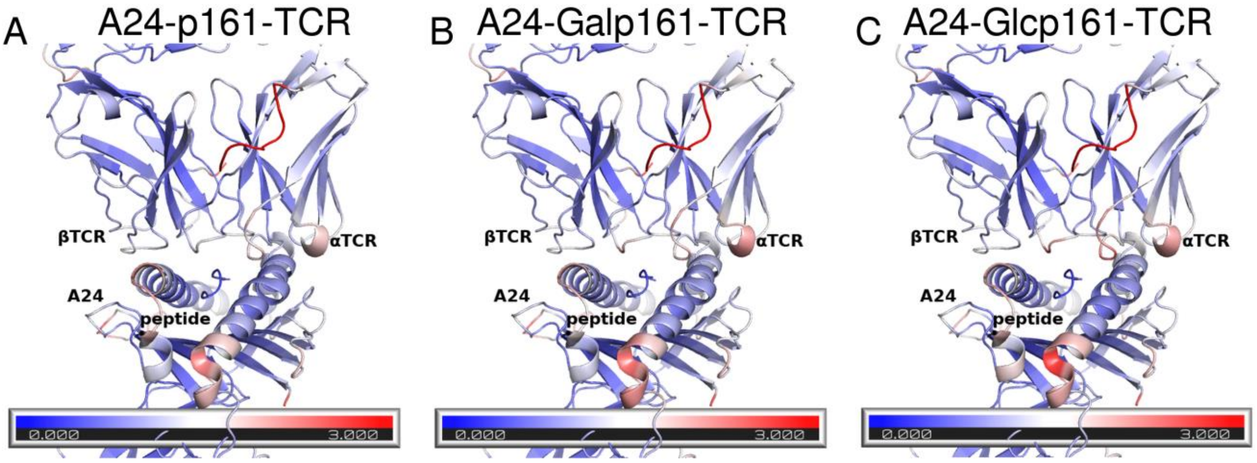

Figure S10. C^{α} RMSF of A24-peptide-TCR complexes: (**A**) p161, (**B**) Galp161, and (**C**) Glcp161 during the last 200 ns of the MD simulations. RMSF values range from very low (blue) to 3 Å (red).

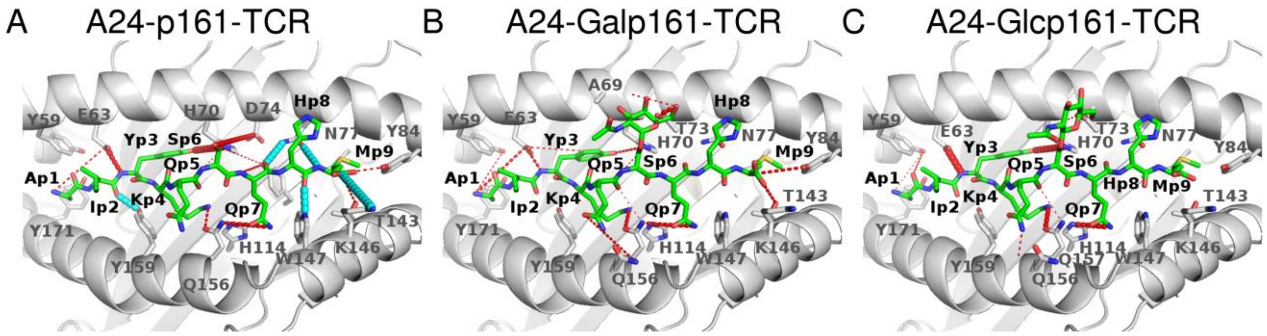

Figure S11. Hbond interactions between A24 and peptide residues in the A24-peptide-TCR complexes, where the peptides are (**A**) p161, (**B**) Galp161, and (**C**) Glcp161 during the 800–1000 ns period of the MD simulations. A24 is in grey ribbons, interacting residues are in sticks, while the peptides are in green sticks

with Oxygen atoms in red and Nitrogen atoms in blue. Hbonds that are present in all three complexes with a lifetime >10% are shown as dashed cyan lines in panel (A). They are not shown in the other panels for clarity. The Hbonds that have lifetimes >10% are in dashed red lines. The thickness corresponds to the average lifetime. In the case of multiple hbond donors and acceptors as in Arg and Asp, the atoms between the donor/acceptor atoms are used to show the hbonds.

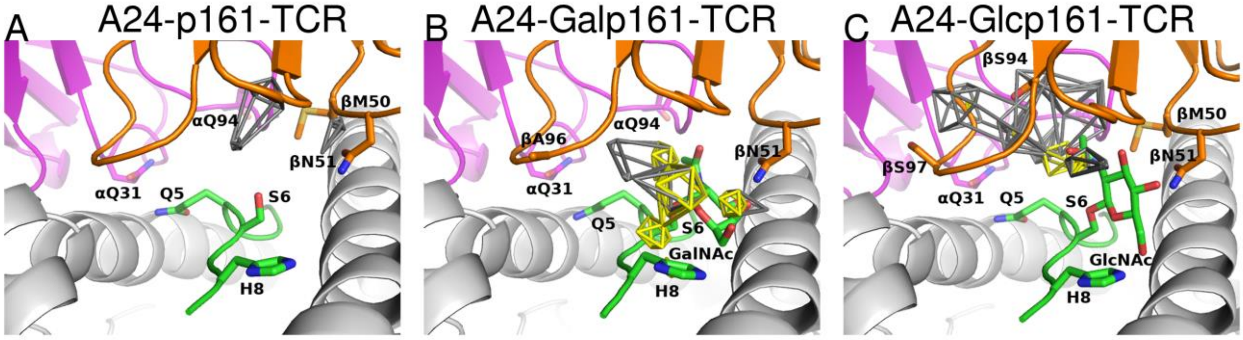

Figure S12. Hbond interactions between the peptides and TCR residues in the MD simulations of the A24-peptide-TCR complexes: (**A**) p161, (**B**) Galp161, and (**C**) Glcp161 (C) during the 800–1000 ns period. A24, peptides, α- and β- chains of TCR are in grey, green, pink, and orange ribbons. respectively. Interacting residues are in sticks, with Oxygen atoms in red and Nitrogen atoms in blue. Residues involving in hbond interactions are in sticks, with Oxygen atoms in red and Nitrogen atoms in blue. Hbonds between peptide and TCR are presented in grey meshes while hbonds between H8 and monosaccharide atoms are presented in yellow meshes. The size of the mesh corresponds to the lifetime of the hbonds.

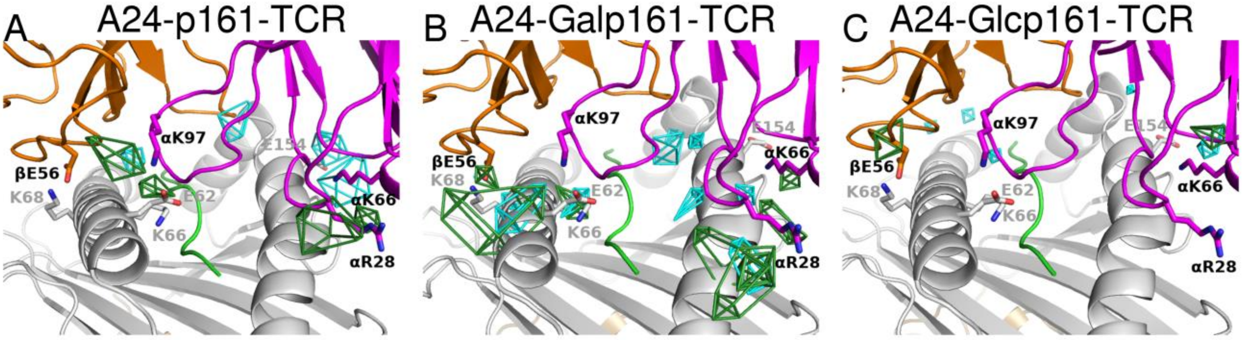

Figure S13. Hbond and salt bridge interactions between A24 and TCR residues in the MD simulations of the A24-peptide-TCR complexes: (**A**) p161, (**B**) Galp161, and (**C**) Glcp161 during the 800–1000 ns period. Peptides, α- and β- chains of TCR are in green, pink, and orange ribbons. Residues involved in salt bridge and hbond interactions are in sticks, with Oxygen atoms in red and Nitrogen atoms in blue. Hbonds are presented as cyan meshes while the salt bridge interactions are presented as green meshes. The size of the mesh corresponds to their lifetimes.
